# Early molecular signatures of responses of wheat to *Zymoseptoria tritici* in compatible and incompatible interactions

**DOI:** 10.1111/ppa.12633

**Published:** 2016-11-22

**Authors:** E. S. Orton, J. J. Rudd, J. K. M. Brown

**Affiliations:** ^1^ John Innes CentreNorwich Research ParkNorwichNR4 7UHUK; ^2^ Rothamsted ResearchHarpendenAL5 2JQUK

**Keywords:** gene expression, MPK3, plant–host interactions, septoria, wheat, *Zymoseptoria tritici*

## Abstract

*Zymoseptoria tritici*, the causal agent of septoria tritici blotch, a serious foliar disease of wheat, is a necrotrophic pathogen that undergoes a long latent period. Emergence of insensitivity to fungicides, and pesticide reduction policies, mean there is a pressing need to understand septoria and control it through greater varietal resistance. *Stb6* and *Stb15*, the most common qualitative resistance genes in modern wheat cultivars, determine specific resistance to avirulent fungal genotypes following a gene‐for‐gene relationship. This study investigated compatible and incompatible interactions of wheat with *Z. tritici* using eight combinations of cultivars and isolates, with the aim of identifying molecular responses that could be used as markers for disease resistance during the early, symptomless phase of colonization. The accumulation of TaMPK3 was estimated using western blotting, and the expression of genes implicated in gene‐for‐gene interactions of plants with a wide range of other pathogens was measured by qRT‐PCR during the presymptomatic stages of infection. Production of TaMPK3 and expression of most of the genes responded to inoculation with *Z. tritici* but varied considerably between experimental replicates. However, there was no significant difference between compatible and incompatible interactions in any of the responses tested. These results demonstrate that the molecular biology of the gene‐for‐gene interaction between wheat and *Zymoseptoria* is unlike that in many other plant diseases, indicate that environmental conditions may strongly influence early responses of wheat to infection by *Z. tritici*, and emphasize the importance of including both compatible and incompatible interactions when investigating the biology of this complex pathosystem.

## Introduction

*Zymoseptoria tritici* (syn. *Mycosphaerella graminicola*,* Septoria tritici*) is the causal agent of septoria tritici blotch, a serious foliar disease of wheat, especially in mild humid temperate regions. Control of *Zymoseptoria* has been based on a combination of fungicide applications and breeding resistant cultivars (Orton *et al*., 2011; Torriani *et al*., 2015). Increasing levels of insensitivity to systemic fungicides, and the adoption of pesticide reduction policies under European legislation (Jess *et al*., 2014), means that there is now greater need than ever to understand and exploit host resistance to the pathogen. Host defences against this pathogen are still not well understood. There are currently 21 qualitative *Stb* resistance genes known (reviewed by Brown *et al*., 2015) that have a phenotype indicative of a gene‐for‐gene relationship (Brading *et al*., 2002). The mechanism of action of these resistance genes is unknown and, as yet, none of them have been cloned.

*Zymoseptoria tritici* is an unusual fungal pathogen in several respects, as it remains extracellular for its entire life cycle and has a long latent period between infection and symptom development. The fungus appears to remain endophytic during the latent period of 10–14 days before entering a necrotrophic phase during which symptom development occurs (reviewed by Orton *et al*. (2011) and Steinberg (2015)). The necrotrophic phase is suppressed during an incompatible interaction (Keon *et al*., 2007; reviewed by Orton *et al*., 2011). Gene‐for‐gene resistance is presumed to involve recognition of the pathogen by the host but the biological basis of the interaction between specific *Stb* genes and avirulent *Z. tritici* strains is not understood at all. Consequently, there is currently no method of classifying interactions between *Z. tritici* and wheat as compatible or incompatible at an early stage of infection, in contrast to the powdery mildew (*Blumeria graminis*) or rust (*Puccinia* spp.) diseases of cereals caused by biotrophic fungi, in which incompatible interactions are characterized by the hypersensitive response (HR) (Boyd *et al*., 1995; Jagger *et al*., 2011). Genotype‐specific resistances in wheat cultivars and avirulences in *Z. tritici* isolates are currently identified by complicated statistical analysis of quantitative data on necrotic symptoms scored 2–4 weeks after infection (e.g. Arraiano & Brown, 2006).

Expression of plant defence‐related and other genes during interactions between biotrophic pathogens and cereal hosts has been extensively studied (Boyd *et al*., 1994a; Coram *et al*., 2008; Bozkurt *et al*., 2010). In powdery mildew, for example, defence gene expression increases in response to the pathogen in both compatible and incompatible interactions, but during an incompatible interaction the response increases over time, whereas in compatible interactions it weakens (Boyd *et al*., 1994b; Moscou *et al*., 2011). Few studies have compared transcriptional responses of cereal hosts during incompatible or compatible interactions with hemibiotrophic or necrotropic pathogens. During interactions between wheat and either *Magnaporthe oryzae* (adapted) or *Magnaporthe grisea* (non‐adapted) isolates, studied using a microarray, a subset of genes was up‐regulated in all interactions. Some defence genes were up‐regulated earlier in the incompatible interaction but later and more strongly in the compatible interactions (Tufan *et al*., 2009), in contrast to what usually occurs in biotrophic interactions. Defence genes were more strongly up‐regulated in compatible than incompatible interactions of wheat with *Pyrenophora tritici‐repentis* (Adhikari *et al*., 2009). The transcriptional changes during ToxA‐ and ToxB‐induced cell death, leading to tan spot disease symptoms, were consistent with responses usually associated with defence against biotrophs (Pandelova *et al*., 2012).

There is some evidence that the switch to necrotrophy and the activation of leaf cell death during successful infection by *Z. tritici* activates similar signalling pathways to those triggered during an HR to biotrophs (Hammond‐Kosack & Rudd, 2008; Deller *et al*., 2011; Yang *et al*., 2013; Rudd *et al*., 2015). The wheat mitogen‐activated protein kinase 3 (*TaMPK3*) transcript and protein accumulated in wheat leaves after infection by a compatible *Z. tritici* isolate immediately preceding symptom development (Rudd *et al*., 2008, 2015; Yang *et al*., 2013), whereas orthologues of TaMPK3 accumulated in incompatible interactions in tobacco in response to infection by *Tobacco mosaic virus* and in tomato in response to *Pseudomonas syringae* pv. *tomato* (reviewed in Meng & Zhang, 2013). However, it was not established if such molecular responses also occur earlier in infection, when leaf tissue exhibits no macroscopic symptoms, or if such responses could be used as indicators of compatibility or incompatibility.

The aim of the experiments reported here was to investigate the molecular basis of early compatible and incompatible interactions of wheat with *Z. tritici*, developing the approach of Ray *et al*. (2003) and Adhikari *et al*. (2007), and thus to find potential early, presymptomatic, markers for resistance or susceptibility. The cultivars studied have resistances conferred by *Stb6* or *Stb15*, the two most common genes for resistance to *Zymoseptoria* in northern European wheat (Arraiano & Brown, 2006). Resistance to *Zymoseptoria* is a quantitative trait, although compatible and incompatible interactions are usually distinct in the cases of *Stb6* (Brading *et al*., 2002) and *Stb15* (Arraiano *et al*., 2007). The accumulation of TaMPK3 protein was analysed in the early stages of both compatible and incompatible combinations of cultivars and isolates, extending the work of Rudd *et al*. (2008), which previously identified increases in transcript, protein and its activity coincident only with the onset of necrotrophic tissue collapse in the later stages of compatible interactions.

The expression of genes that are normally up‐regulated in resistant responses to biotrophic pathogens was studied to test if they are up‐regulated during a susceptible response to *Z. tritici* (Deller *et al*., 2011). Comparisons were also made using genes assessed in previous studies (Ray *et al*., 2003; Adhikari *et al*., 2007; Shetty *et al*., 2009). The genes included in this study were selected to represent a wide range of features of defence responses that are differentially regulated in host–pathogen interactions, including gene‐for‐gene relationships: *PR‐1* and *lipoxygenase* (Ray *et al*., 2003); *β‐1*,*3‐glucanase* (Shetty *et al*., 2009); *chitinase* (Bolton *et al*., 2008); *peroxidase* and *PAL* (phenylalanine ammonia lyase; Adhikari *et al*., 2007). *Chlorophyll a/b binding precursor* (Rudd *et al*., 2015) was selected as a marker of the initiation of leaf senescence. The *cysteine protease* gene, homologous to *Arabidopsis thaliana Sag12* (Lohman *et al*., 1994) was selected as another marker for senescence. *Mlo* is required for susceptibility to the biotrophic pathogen *B. graminis* but reduces susceptibility to non‐biotrophic fungal pathogens (reviewed by Brown & Rant, 2013). TaMPK3 protein accumulates preceding symptom development (Rudd *et al*., 2008). A protein disulphide isomerase (*PDI*) was selected as it was up‐regulated in two resistant wheat cultivars inoculated with *Z. tritici* in previous studies (Ray *et al*., 2003).

## Materials and methods

### Fungal isolates

The *Z. tritici* isolates used throughout the experiments were IPO323 and IPO88004. The isolates were stored at −80 °C. Spores for plant inoculation were grown on YPD+ agar plates (2% Bacto agar, 2% peptone, 1% yeast extract, 2% glucose at pH 5.8) for 4–7 days at 18 °C with near‐UV light at 350 nm. Spore concentration was estimated using a modified Fuchs Rosenthal counting chamber (Hawksley).

### Plant material

In these experiments, four cultivars possess *Stb6*: Arina, Flame, Poros and Cadenza; Arina and Flame express resistance to the avirulent isolate, IPO323, more strongly than Poros and Cadenza (Arraiano & Brown, 2006). Cultivars Longbow and Avalon are susceptible to IPO323. Longbow and Courtot were used to test the interaction with IPO88004; Longbow possesses *Stb15* and is resistant to this isolate, whereas Courtot is susceptible. Plants used in gene expression experiments with IPO323 were grown in glasshouses set at 18 °C for 16 h (light) and 12 °C for 8 h (dark) with additional lighting for the 16 h period, although external weather conditions caused temperature fluctuations. Plants used in gene expression experiments with IPO88004 and in all experiments for TaMPK3 accumulation were grown in a growth room with temperatures set to 18 °C for 16 h (light) and 12 °C for 8 h (dark). For plant inoculation, the second leaves of 17‐day‐old seedlings were attached, adaxial side up, to Perspex sheets using double‐sided tape (Keon *et al*., 2007). The leaves were inoculated evenly with a fungal spore solution at a density of 10^7^ spores per mL of water containing 0.1% (v/v) Tween 20 (Sigma‐Aldrich) using a swab stick with a cotton sterile tip (Fisher Scientific). Plants inoculated with *Z. tritici* were placed in trays with plastic lids and under black plastic sheeting, to achieve dark conditions with high relative humidity, for 48 h. After this time, the plants were moved into the light but remained in high humidity conditions.

### Experimental design

In all experiments the cultivars Longbow, Flame, Avalon, Cadenza, Arina and Poros were inoculated with the isolate IPO323. The cultivars Longbow and Courtot were inoculated with isolate IPO88004. Control leaves were mock inoculated with water containing 0.1% Tween 20. In all experiments, some plants were kept for up to 21 days to check for pycnidium formation in compatible interactions. If pycnidia did not develop by 21 days on susceptible cultivars the replicate was not used for further analysis.

### Quantification of TaMPK3 protein

Analysis of the amount of TaMPK3 protein that accumulates in each cultivar tested was carried out using western blots. For each of two replicates, samples were collected at 1, 3, 7, 10, 11, 14, 15, 16 and 17 days after inoculation (dai) and mock‐inoculated samples taken at 1, 10 and 17 dai as controls. At each time point three leaves were collected and pooled as one sample for each cultivar/isolate/time point combination. This experiment was carried out independently of the experiment to quantify gene transcription.

Leaves were collected directly into liquid nitrogen from each cultivar/isolate combination and stored at −80 °C before protein extraction was carried out. Protein was extracted by grinding frozen cells in extraction buffer (37.5 mm Tris‐HCl pH 7.4, 112.5 mm NaCl, 22.5 mm EGTA, 0.15% v/v Tween 20, 1.5 mm NaF, 0.75 mm sodium molybdate, 1.5 mm DTT, 0.75 mm PMSF, 15 μg mL^−1^ leupeptin, 15 μg mL^−1^ aprotinin, 22.5 mm *β*‐glycerophosphate) followed by centrifugation at 23 000 ***g*** for 20 min at 4 °C (Rudd *et al*., 2008).

A Bradford assay was performed to quantify the concentration of protein in each sample using Bradford protein assay reagent (Bio‐Rad) and comparing with a bovine serum albumin standard of 2 mg mL^−1^ (Sigma‐Aldrich). Readings were taken using a BioPhotometer (Eppendorf) at 595 nm, the wavelength at which the bound form of the reagent is absorbed. Samples were mixed with a loading dye consisting of 5% v/v 2‐mercaptoethanol, 250 mm Tris‐HCl pH 6.8, 10% w/v SDS, 30% v/v glycerol, and bromophenol blue, so that all samples contained an equal amount of protein. Samples were stored at −20 °C. Samples were heated to 90 °C for at least 5 min to solubilize the protein and then centrifuged for 5 min at 16 100 ***g*** before being loaded onto the gel.

Approximately 120 μg of protein was separated on 10% SDS‐PAGE gels and tank blotted onto Hybond ECL nitrocellulose membrane (GE Healthcare Life Sciences) using the Mini Trans‐Blot cell (Bio‐Rad) following the manufacturer's protocol. Membranes were blocked overnight at 4 °C in Tris‐buffered saline (TBS)‐Tween (20 mm Tris‐HCl pH 7.3, 137 mm NaCl, 0.1% v/v Tween 20) with 5% skimmed milk powder. The MAPK‐specific antibody TaMPK3‐N (affinity purified; Rudd *et al*., 2008) at 1:500 dilution in TBS‐Tween with 5% milk powder was incubated with the membranes at room temperature for 90 min. After the membranes were washed five times with TBS‐Tween, chemiluminescent detection using Amersham ECL Plus Western Blotting Detection Reagents was carried out in accordance with the manufacturer's instructions (GE Healthcare Life Sciences).

### Quantification of gene transcription

Transcription of 11 genes of interest (Table 1) was analysed in all the above cultivar/isolate combinations using quantitative reverse transcription PCR (qRT‐PCR). The experiment was replicated three times. In each replicate, three leaves from each cultivar/isolate combination were collected and pooled as one sample for each interaction at 0.5, 1, 3, 7, 10 and 14 dai. Mock‐inoculated leaves were also sampled at each time point as controls.

**Table 1:** List of the genes of interest (GoI) and reference (Ref) genes

Gene	Gene name	GenBank accession no.	Primer (5′–3′)		Reference
GoI	*β‐1,3‐glucanase* (*β‐glu*)	Y18212.1	L	AACGACCAGCTCTCCAACAT	Shetty *et al*. (2009)
			R	GTATGGCCGGACATTGTTCT	
GoI	*Chitinase 2* (*Chit*)	CD490414	L	GAGCAGCCTCACTTGCTAGG	Bolton *et al*. (2008)
			R	ATACGCATGCCGAACGTTTA	
GoI	*Chlorophyll a/b binding precursor*	U73218.1	L	CCTTGGTGAGGCCCGAGTCACTAT	J. J. Rudd (unpublished)
			R	TTGGCAAAGGTCTCGGGGTC	
GoI	*Cysteine protease* (*Sag12*)	CA680100	L	GTTCTCGGACCTCACCAGCGAA	J. J. Rudd (unpublished)
			R	ACGCCCACCAACAACCGCAT	
GoI	*Lipoxygenase* (*Lox*)	AY253443	L	GGGCACCAAGGAGTACAAGGA	Ray *et al*. (2003)
			R	CGATCACCGACACTCCAATG	
GoI	*Mlo*	CA745732	L	CCTACCACTATACGCCGTCGTCTCC	J. J. Rudd (unpublished)
			R	CACCGACGAGTTTGCCCGTGTAT	
GoI	*TaMPK3*	AF079318.1	L	TACATGAGGCACCTGCCGCAGT	Rudd *et al*. (2008)
			R	GGTTCAACTCCAGGGCTTCGTTG	
GoI	*Peroxidase* (*Perox*)	X85229	L	CCAGCACGACACGTGAATG	Adhikari *et al*. (2007)
			R	CATGATTTGCTGCTGCTCGTA	
GoI	*Phenylalanine ammonia lyase (PAL)*	AY005474	L	GTGTCTCCATGGACAACACCCG	Adhikari *et al*. (2007)
			R	TCAATGGCCTGGCACAGAGC	
GoI	*PR‐1*	AY258615.1	L	ACGTACGCCAACCAGAGGATCA	Ray *et al*. (2003)
			R	GCATGCGATTAGGGACGAAAGAC	
GoI	*Protein disulphide isomerase* (*PDI*)	AF262980	L	TTATGACTTTGGCCACACCG	Ray *et al*. (2003)
			R	CGAGCTCATCAAATGGCTTG	
Ref	*Ta Elongation factor*	M90077.1	L	TGGTGTCATCAAGCCTGGTATGGT	Coram *et al*. (2008)
			R	ACTCATGGTGCATCTCAACGGACT	
Ref	*Hv GapDH*	M36650	L	CCTTCCGTGTTCCCACTGTTG	McGrann *et al*. (2009)
			R	ATGCCCTTGAGGTTTCCCTC	
Ref	*Ta Ubiquitin*	M60175	L	CCTTCACTTGGTTCTCCGTCT	Rostoks *et al*. (2003)
			R	AACGACCAGGACGACAGACACA	

Leaves were cut off the plants, placed immediately in liquid nitrogen then stored at −80 °C before further processing. Total RNA was isolated from frozen leaf tissue using either the Tri‐reagent procedure (Sigma‐Aldrich), following the manufacturer's protocol and using the additional step suggested for polysaccharide‐containing tissues (for experiments using isolate IPO323), or the RNeasy Plant Mini kit (QIAGEN; for experiments using isolate IPO88004), following the manufacturer's instructions.

A DNase treatment was carried out on the extracted RNA using the TURBO DNA‐free kit (Ambion), following the manufacturer's ‘rigorous’ procedure, which is designed to remove DNA from samples containing >2 μg DNA per 50 μL RNA. To test if all genomic DNA had been removed from the RNA sample, each sample was subjected to qRT‐PCR analysis for 40 cycles using a set of primers designed for cDNA (Table 1, reference primers). The total quantity of RNA was quantified using a Picodrop100 spectrophotometer (Picodrop Ltd). One microgram of total RNA was converted to cDNA using Superscript III reverse transcriptase (Invitrogen) using random hexamers, following the manufacturer's protocol.

Each cDNA sample was diluted 1:20 in nuclease‐free water. qRT‐PCR was performed using a CFX96 detection system (Bio‐Rad), in plates with optically clear seals (both Thermo Scientific). Each reaction contained 5 μL diluted cDNA and 12.5 μL Brilliant II SYBR Green master mix (Agilent Technologies), with 500 nm each of the left and right primers in a total volume of 25 μL. All PCRs were carried out using the following cycle: 95 °C for 10 min; then 40 cycles of denaturation at 95 °C for 30 s, annealing at 56 °C for 30 s and extension at 72 °C for 30 s. Immediately after this, a melt curve analysis was carried out by ramping from 65 to 90 °C. All samples had two technical repetitions. Primer efficiencies for the genes of interest were tested for each primer pair using a dilution series from 1:10 to 1:10 000 made from a mixture of cDNA samples. Amplification values ranged from 1.89 to 2.15.

### Gene transcription data analysis

Quantification cycle (*C*_q_) values of three reference genes (Table 1) were checked for stability using genorm software (Vandesompele *et al*., 2002; https://genorm.cmgg.be/). The reference genes in all experiments were found to be stable. −*C*_q_ is directly proportional to the logarithm of the amount of cDNA in the sample, hence calculations on *C*_q_ are equivalent to those on log[cDNA]; in particular, the arithmetic mean of −*C*_q_ is equivalent to the geometric mean of [cDNA]. *C*_q_ of the reference genes was standardized to avoid large but uninteresting differences between the mean level of transcription of each reference gene. The three reference genes were treated as replicates. *C*_q_ of each reference gene was standardized to the grand mean of all reference genes to give *C*_q,std_. A general linear model (GLM) was fitted to the *C*_q,std_ data including the factors Isolate, Cultivar, Compatibility, Time, Treatment, Gene and Replicate and combinations of those factors (some of these factors are partially confounded so interactions between them could not be fitted). After successive elimination of terms that were not statistically significant (*P *>* *0.05), the final model was (Isolate + Compatibility + Cultivar)*(Time*Treatment + Gene) + Isolate∣Replicate + (Time·Gene)∣(Isolate+Cultivar) + (Treatment·Gene)∣(Time*Replicate), where * is the crossing operator (the sum of relevant main effects plus interactions), ∣ is the nesting operator (main effect of the factor on the left plus interactions with factors on the right) and · is the interaction operator. The Replicate term refers to the six inoculations done as biological replicates in the series of experiments (Replicates 1–3 involved IPO323 and 4–6 involved IPO88004). Treatment was either inoculation with *Z. tritici* or mock‐inoculation. The Gene factor classified genes studied either as one of the set of reference genes or, individually, a gene of interest. Compatibility was the compatible or incompatible response of the cultivar/isolate combination.

Effects involving the term Treament·Gene are relevant to biological interpretation of the *C*_q,std_ data in terms of differences in the expression of test and reference genes in inoculated and mock‐inoculated leaves. Predicted means were calculated for each combination of Treatment with Cultivar and Gene at each Time.

The effect of infection by *Z. tritici* on gene transciption was calculated from predicted mean *C*_q,std_ values. For each gene, *C*_q,std_ is proportional to the logarithm of the quantity of cDNA in the sample to the base of the amplification value. First, *C*_q_ for the target gene in an inoculated sample (*C*_q,ti_) was standardized by comparing it to the mean *C*_q_ for the reference genes in that sample (*C*_q,ri_)_._ Likewise, *C*_q_ for the target (*C*_q,tm_) and reference (*C*_q,rm_) genes were calculated for the relevant mock‐inoculated sample. The four *C*_q_ values were estimated separately and the logarithm of the fold increase in gene expression was proportional to (*C*_q,ti_ − *C*_q,tm_) − (*C*_q,ri_ + *C*_q,rm_) = Δ*C*_q_. The standard error of Δ*C*_q_ was calculated from the variance–covariance matrix of the predicted means as the square root of the sum of the squared standard errors of the four *C*_q_ estimates. Calculation of Δ*C*_q_ by the method described here is based on the assumption that all genes have the same amplification values. This is approximately correct because the amplification values for the 11 genes varied within a narrow range from 1.89 to 2.05. While comparisons between treatments of Δ*C*_q_ for the same target gene are exact, comparisons involving different target genes are approximate.

## Results

**Figure 1:**
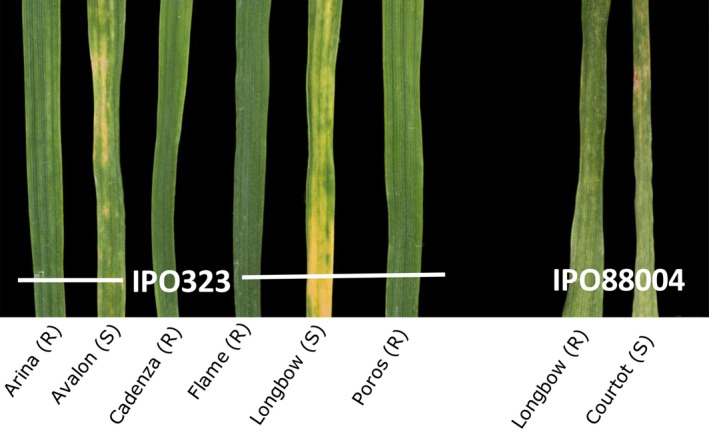
Phenotypes of compatible and incompatible interactions between wheat and *Zymoseptoria tritici* used in this study. The eight cultivar–isolate combinations were used throughout the experiments. Avalon and Longbow were susceptible to isolate IPO323 and showed necrotic symptoms at 14 days after inoculation (dai). Courtot was susceptible to isolate IPO88004 and showed necrotic flecks at 14 dai. Pycnidia were produced subsequently in these interactions. Arina, Cadenza, Flame and Poros carry the *Stb6* resistance gene and are resistant to isolate IPO323, Longbow has the resistance gene *Stb15* and is resistant to IPO88004; although it showed some necrotic flecking, pycnidia did not develop.

The time points at which the samples were taken were chosen to cover the period from inoculation to the onset of necrosis, to focus on the early processes that determine whether or not the host–parasite interaction between wheat and *Z. tritici* is compatible. Figure 1 shows symptoms when the final samples were collected on day 14 after inoculation. No symptoms were visible at earlier time points. The cultivars Arina, Cadenza, Flame and Poros, which were resistant to IPO323, remained green throughout the course of the experiment. Longbow was resistant to IPO88004 although it showed some necrosis at 14 dai, but never any pycnidium development.

**Figure 2:**
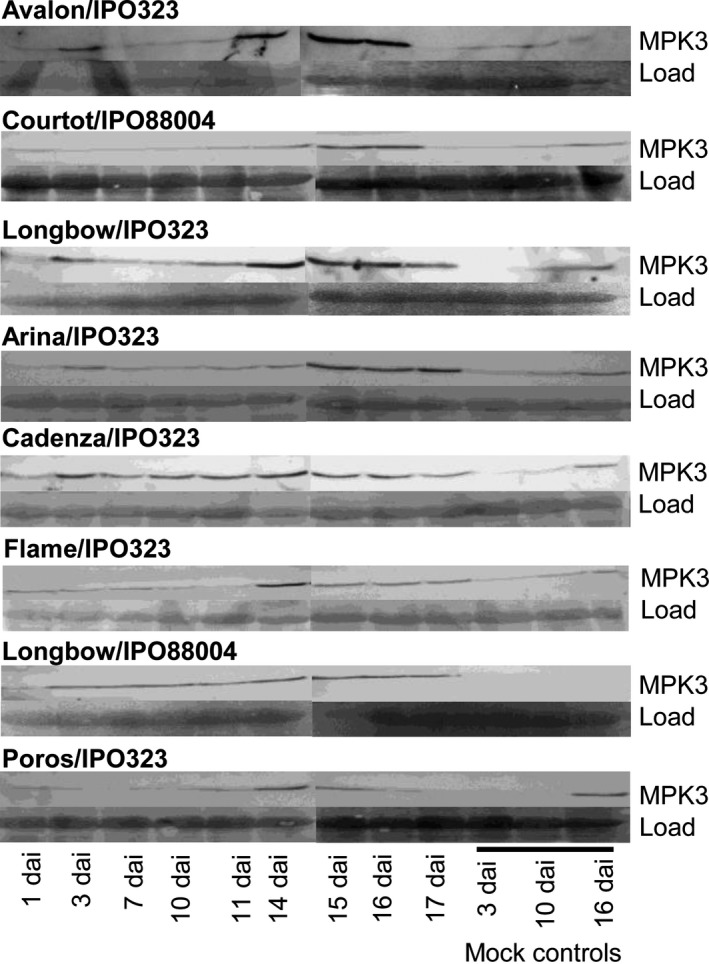
Accumulation of TaMPK3 during compatible and incompatible interactions between wheat and *Zymoseptoria tritici*. Changes in TaMPK3 levels over a 17 day period after inoculation with *Z. tritici* are shown by western blots probed with a TaMPK3‐specific antibody for eight different cultivar/isolate combinations: the interactions Longbow–IPO323, Avalon–IPO323 and Courtot–IPO88004 were compatible, the other combinations were incompatible. The time course is shown over two blots. Note the comparisons between mock‐inoculated and *Z. tritici*‐inoculated samples at 3, 10 and 16 days after inoculation (dai). Protein loading levels are shown for each blot in the 60‐kD region using amido black staining. TaMPK3 accumulated in all cultivars inoculated with *Z. tritici*, independent of compatibility; some TaMPK3 also accumulated in the mock‐inoculated controls at 10 and 16 dai. Gels from a replicate experiment with the samples in a different order are shown in Figure S1.

Western blots indicated that TaMPK3 accumulated to some extent in all cultivars in both compatible and incompatible interactions at all time points (Figs 2 & S1). In most cases at 3 and 10 dai, TaMPK3 levels were similar in mock‐inoculated samples as in the corresponding samples inoculated with *Z. tritici*. In all treatments, levels of TaMPK3 remained fairly constant up to 10 dai but from 11 dai onwards, TaMPK3 levels in samples inoculated with *Z. tritici* varied, but not in a way that was clearly consistent with the compatibility of the cultivar/isolate combination. Crucially, at 16 dai TaMPK3 accumulated to higher levels in inoculated than mock‐inoculated samples in compatible interactions (Longbow and Avalon with IPO323 and Courtot with IPO88004) whereas TaMPK3 levels were generally similar in incompatible interactions as in the mock treatment.

### Gene transcription

Expression of genes involved in plant defence, cell death and senescence were studied in compatible and incompatible interactions of wheat cultivars with *Z. tritici* isolates by qRT‐PCR. The level of mRNA in wheat leaves was measured in terms of quantification cycles (*C*_q_) and the variate analysed was *C*_q,std_; each reference gene was standardized by setting its mean *C*_q_ over all treatments to be equal to the grand mean of all three reference genes, to give the variate *C*_q,std_. This prevented comparisons involving the reference genes from being dominated by systematic variation between them, which is irrelevant to the aim of the experiments reported here. A GLM of *C*_q_ data was used to investigate factors that affected gene expression in plants inoculated with *Z. tritici*. Four measurements of gene expression are relevant here: *C*_q_ of a gene of interest in inoculated (*C*_q,gene,inoc_) and mock‐inoculated leaves (*C*_q,gene,mock_), and the average *C*_q_ of a set of reference genes in both types of leaf (*C*_q,ref,inoc_, *C*_q,ref,mock_). The expression (*C*_q,gene,inoc_ − *C*_q,gene,mock_) − (*C*_q,ref,inoc_ − *C*_q,ref,mock_) compares the expression of the gene of interest to that of reference genes in inoculated leaves in relation to mock‐inoculated leaves. The relevant term in the GLM is the Treatment·Gene interaction. Only terms containing this interaction are discussed here.

**Figure 3:**
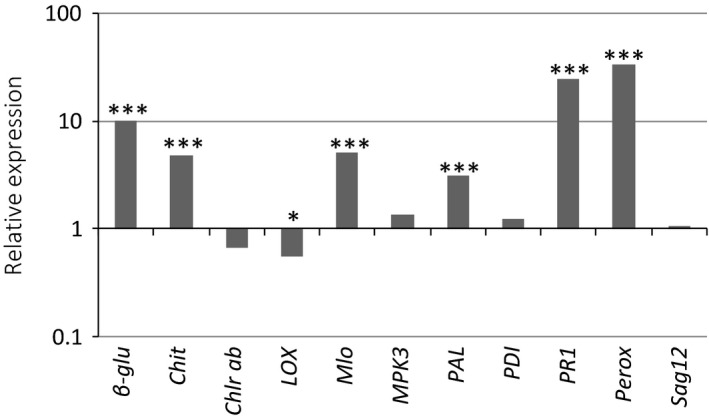
Expression of the genes of interest during the interaction between wheat and *Zymoseptoria tritici*. The relative expression of genes of interest is shown in comparison to the mock‐inoculated controls as obtained by qRT‐PCR. Data for all cultivar/isolate combinations and for all time points tested has been combined. *β‐1*,*3‐glucanase*,* chitinase*,* Mlo*,*PAL*,*PR1* and *peroxidase* were very significantly up‐regulated (***P <* *0.001) and *lipoxygenase* was significantly down‐regulated (*0.05 > *P *>* *0.01) where *P* is the significance of differences of relative expression from 1.

The main effect of Treatment·Gene was much more significant than any interaction involving Treatment·Gene (Table 2), regardless of whether the interaction was compatible or incompatible. This implies that the genes studied here were expressed or repressed in response to *Z. tritici* infection as such, and that modulation of their expression by their interaction with the host in the first 2 weeks after inoculation was comparatively minor. The genes with the highest overall differential expression compared to the reference genes were *PR1* and *peroxidase*, followed by *β‐1*,*3‐glucanase*. *Chitinase*,* Mlo* and *PAL* were also significantly up‐regulated over time. Overall, *chlorophyll a/b binding precursor* and *lipoxygenase* were weakly down‐regulated and *cysteine protease* (*Sag12*) showed little differential expression. *MPK3* and *PDI* showed no significant alteration in expression compared to mock‐inoculated samples (Fig. 3).

**Table 2:** Reduced anova table showing significant terms (nonsignificant terms with *P *>* *0.05 have been removed)

	d.f.	MS	VR	*F* pr.
Treatment·Gene	11	147.0	37.4	<0.001
Time·Treatment·Gene	55	8.3	2.1	<0.001
Replicate·Treatment·Gene	103	16.3	4.1	<0.001
Replicate·Time·Treatment·Gene	535	6.8	1.7	<0.001
Residual	2661	3.9		
Total	3992	133.3		

d.f., degrees of freedom; MS, mean squared deviations (variance); VR, variance ratio. Residual term: replicate leaves given exactly the same combination of factors.

The final model fitted was (Isolate + Compatibility + Cultivar)*(Time*Treatment + Gene) + Isolate∣Replicate + (Time·Gene)|(Isolate+Cultivar) + (Treatment·Gene)∣(Time*Replicate). Only effects involving the Treatment·Gene interaction are shown, indicating a difference between inoculated and mock‐inoculated plants in the expression of the genes of interest relative to a set of reference genes. The analysis is from qRT‐PCR data of gene expression of 11 wheat genes of interest on eight cultivar/*Zymoseptoria tritici* isolate combinations over six time points: 0.5, 1, 3, 7, 10 and 14 days after inoculation, prior to onset of pycnidial development. The variate analysed is *C*_q,std_, which is each reference gene standardized to the grand mean of all reference genes.

The Cultivar, Isolate and Compatibility terms had no significant interaction with the Treatment·Gene effect. This implies that there were no significant differences between cultivars’ responses to inoculation and that overall the expression of the genes of interest in resistant cultivars was similar to susceptible cultivars. It also implies that the isolates used did not have a significant differential effect on gene expression and there was no significant evidence for a distinct response in either a compatible or an incompatible interaction.

**Figure 4:**
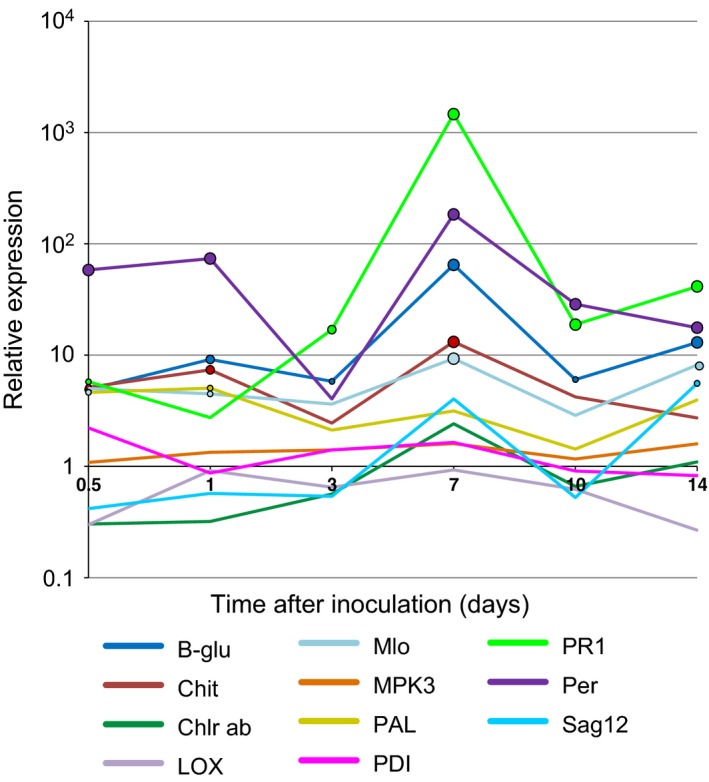
Expression of the genes of interest during the interaction between wheat and *Zymoseptoria tritici* between 0.5 and 14 days after inoculation (dai). Expression of *β‐1*,*3‐glucanase*, c*hitinase*,* chlorophyll a/b binding precursor*,* cysteine protease* (*Sag12*), *lipoxygenase*,* Mlo*,*MPK3*,*PAL*,*PR1*,* peroxidase* and *PDI* was tested compared to mock‐inoculated controls at 0.5, 1, 3, 7, 10 and 14 dai by qRT‐PCR on eight cultivar/isolate combinations. During analysis, the compatibility, cultivar and isolate factors were found not to be statistically significant, therefore all cultivars and isolate combinations have been combined. Significance of differences of relative expression from 1 is indicated by the size of circles (large circles: *P *<* *0.001; medium circles: 0.01 > *P *>* *0.001; small circles 0.05 > *P *>* *0.01).

There was relatively minor, but statistically significant, variation in the expression of genes of interest over the course of the infection (comparing the size of the Time·Treatment·Gene effect to the Treatment·Gene interaction in Table 2). There was no significant variation between different variety/isolate combinations over the time course. For the majority of genes, there was a trend for expression to peak at 7 dai, followed by a reduction then a further increase at 14 dai (Fig. 4). At 7 dai, the expression levels of *β‐1*,*3‐glucanase*,* chitinase*,* PR1*,* peroxidase* and *Mlo* were significantly greater than in the mock‐inoculated plants (*P *<* *0.001). *PR1* and *peroxidase* were also strongly up‐regulated at 10 and 14 dai (*P *<* *0.001), *peroxidase* at 0.5 and 1 dai (*P *<* *0.001), and *β‐1*,*3‐glucanase* at 10 dai (*P *<* *0.05) and 14 dai (*P *<* *0.001). *Sag12* and *Mlo* were significantly but weakly up‐regulated at 14 dai (*P *<* *0.05 and *P *<* *0.01 respectively; Figs 4 & S4).

Analysis of gene expression across the replicates showed there was minor, but statistically significant, variation in gene expression between replicates (compare the Replicate·Treatment·Gene effect to Treatment·Gene in Table 2). *PR1*,* peroxidase* and *β‐1*,*3‐glucanase* showed the greatest variation across replicates, with the greatest up‐regulation seen in replicate 2 (Fig. S2). The former two genes also had the highest relative expression of all those tested. *Chitinase* and *Mlo* were also significantly more up‐regulated in replicate 2 than average, but not in any other replicates. The expression of *chlorophyll a/b binding precursor* was slightly but significantly lower in replicate 5 than in the other replicates.

In addition to variation between replicates for many of the genes of interest, there was also variation in the expression levels of genes at each time point between the biological replicates (Replicate·Time·Treatment·Gene term in Table 2, *P* < 0.001; Fig. S3). The most consistent feature was the up‐regulation of five genes, *β‐1*,*3‐glucanase*,* chitinase*,* Mlo*,* PR1* and *peroxidase*, in all six replicates at 7 dai. This was closely followed by the up‐regulation of four genes (*chitinase*,* Mlo*,* PR1* and *peroxidase*) at 14 dai.

## Discussion

Expression of most of the genes reported here and accumulation of TaMPK3 protein responded to infection by *Z. tritici* in its early, presymptomatic stages. Inoculating the plants with *Z. tritici*, irrespective of the host and pathogen genotypes, caused differential regulation of 7 of the 11 genes tested compared to mock‐inoculated control plants. The genes investigated were representative of a wide range of processes involved in plant defence against parasites and responses to abiotic stress. Early responses of wheat to infection by *Z. tritici* therefore involve similar pathways to those triggered by many other pathogens but the pattern of effects reported here leads to three broad conclusions relevant to research on septoria tritici blotch.

In all the processes reported here, whether expression of defence‐related and stress‐related genes, or TaMPK3 protein accumulation, there was no significant difference between isolates, cultivars, and compatible or incompatible interactions in the presymptomatic period of infection by *Z. tritici*. The first conclusion, therefore, is that there is no evidence that the defences induced by qualitative septoria‐resistance genes are the same as those in other diseases in which there are gene‐for‐gene relationships. The lack of variation between compatible or incompatible interactions is particularly striking because the experimental design was capable of detecting statistically significant variation in gene expression over time or in different biological replicates. In other plant diseases, there are distinct molecular responses of the host to infection by virulent or avirulent pathogen genotypes, including expression of several of the genes reported here in diseases caused by biotrophic (Boyd *et al*., 1994a,b; Bolton *et al*., 2008; Coram *et al*., 2008; Bozkurt *et al*., 2010) and non‐biotrophic pathogens (*M. oryzae*: Tufan *et al*., 2009; *P. tritici‐repentis*: Adhikari *et al*., 2009). The absence of such variation here is consistent with the view that *Z. tritici* has a mode of infection that differs markedly from that of other well‐studied pathogens (Hammond‐Kosack & Rudd, 2008) and indicates that mechanisms of gene‐for‐gene resistance to *Zymoseptoria* differ from those in other diseases.

TaMPK3 protein accumulated very early in the infection process, within 1 dai in almost all samples, in all the interactions tested irrespective of the cultivar or isolate, but did not discriminate incompatible and compatible interactions in early, presymptomatic infection up to 10 dai. At 3 and 10 dai, TaMPK3 accumulated in the mock‐inoculated samples to a similar level as in corresponding samples inoculated with *Z. tritici*, implying that at these earlier time points TaMPK3 production may have been a response to the inoculation procedure or the environmental conditions rather than a response to the fungus or to a specific type of interaction between wheat and *Z. tritici*. The authors are not aware of evidence that genotypes of wheat or any other plant vary in levels of MPK3 before exposure to stress. The results are consistent with MPK3 being induced in response to wounding or during leaf senescence. MPK3 is implicated in the onset of lesion formation during compatible interactions with non‐biotrophic pathogens (Xiong & Yang, 2003), the development of an HR in response to biotrophic pathogens and in responses to stress and biotrophic pathogens (Meng & Zhang, 2013). The function of MPK3 in plant defence appears to involve a complex signalling network that can be modulated through interplay of network components affecting both SA‐ and JA‐responsive genes (Meng & Zhang, 2013). Rudd *et al*. (2008) found accumulation of TaMPK3 just before and during the onset of necrotic symptoms in compatible interactions, which is consistent with greater production of TaMPK3 in compatible interactions compared to mock‐inoculated plants at 16 dai (Figs 2 & S1). This, as well as the lack of additional accumulation of TaMPK3 in incompatible interactions in most samples compared to the mock treatment, is consistent with MPK3 being associated with necrotic lesions in non‐biotrophic diseases, particularly septoria tritici blotch.

The genes with the strongest difference in expression between inoculated and mock‐inoculated plants, *β‐1*,*3‐glucanase*,* chitinase*,* PR1*,* Lox*,* Mlo*,* PAL* and *peroxidase*, were differentially expressed across the time course of infection. All except *Lox* were up‐regulated in both compatible and incompatible interactions with *Z. tritici* so these are likely to be involved in general responses to the presence of the fungus, rather than a virulent isolate specifically. *Lox* was the only gene consistently down‐regulated; again, there was no distinction between compatible and incompatible interactions. *TaMPK3* was not significantly altered in expression during these experiments, nor were *PDI*, previously reported as being induced in a resistant variety in response to *Z. tritici* (Ray *et al*., 2003), *chlorophyll a/b binding precursor*, a stress‐induced gene, and *SAG12*, despite the latter two genes being induced during foliar senescence (Lohman *et al*., 1994).

The distinct peak in expression of several genes at 7 dai suggests that, at this time, the fungus triggers a general defence response by the host, despite the lack of evidence for differentiation in the development of *Z. tritici* on resistant and susceptible hosts at this time (Shetty *et al*., 2003). Rudd *et al*. (2015) measured responses at 9 dai of a susceptible host during the onset of lesion development and also found strong up‐regulation of *PR1*,* peroxidase* and *β‐1*,*3‐glucanase*. The smaller peak at 14 dai might correspond to the switch to the necrotrophic phase. The lack of differentiation between responses of resistant and susceptible cultivars demonstrates that, up to 14 dai, plant defences are not specific to an incompatible isolate.

Some genes tested here have previously been reported to be differentially regulated between compatible and incompatible responses to *Z. tritici* (*PAL* and *LOX*: Adhikari *et al*., 2007; *PDI*: Ray *et al*., 2003; *TaMPK3*: Rudd *et al*., 2008) but were not differentially regulated here. Levels of *chitinase* expression have varied between studies; the results of the current study are consistent with Shetty *et al*. (2009) but differ from Adhikari *et al*. (2007) who found that very little transcript accumulated during compatible interactions. *PR1* also has different patterns of expression between studies; in the current experiments, both susceptible and resistant cultivars accumulated *PR1*. Ray *et al*. (2003) also found that *PR1* was up‐regulated strongly in both compatible and incompatible interactions with *Z. tritici* at 12 hai (their study did not include later time points at the start of macroscopic symptom development). In contrast, Rudd *et al*. (2015) found early down‐regulation and late accumulation of *PR1* amongst a large number of other defence‐associated genes in a compatible interaction using RNA‐seq technology. Adhikari *et al*. (2007) demonstrated strong induction of *PR1* in incompatible interactions but little *PR1* expression in susceptible cultivars. The second broad conclusion, therefore, is that expression of defence‐related and stress‐related genes in wheat in response to *Z. tritici* may depend not only on the host and parasite genotypes but also on environmental conditions such as the methods of growing and infecting wheat plants.

In addition to the differences between studies, there has also been high variability in gene expression within studies. The large differences between replicate tests in the experiments presented here are comparable to results of other experiments (Ray *et al*., 2003; Adhikari *et al*., 2007). The replicates of experiments presented here were carried out at different times and therefore will have been subject to differing environmental conditions that could have influenced the expression of host defences. Environmental conditions could also explain differences seen between replicates in other studies and between different studies.

High variability in defence gene expression may be inherent in this pathosystem. There is a long latent period in which there is little accumulation of fungal biomass within the leaf (Keon *et al*., 2007) so pathogen development may not happen synchronously throughout a leaf. Gene expression may therefore be less uniform than in diseases in which there is synchronous pathogen development, such as barley powdery mildew (Boyd *et al*., 1994b). Furthermore, the high inoculum density used in this and other studies may affect the penetration ability of the fungus or its ability to spread within the apoplast (Fones *et al*., 2015) and thus the expression of host defences.

A further factor that has not been considered either in these experiments or in previous work on *Z. tritici* or most other pathogens is that expression of defence‐related genes may be in part due to responses to microflora that are able to use the infection progress of *Z. tritici* to their own advantage and it may be the microbe‐associated molecular patterns (MAMPs) associated with these that trigger a defence response. Boyd *et al*. (1994b) demonstrated that, in response to wounding, defence‐related genes were either less up‐regulated or not expressed at all in barley grown in sterile conditions, compared to plants that had been grown and either wounded or inoculated with *B. graminis* in normal conditions. This implied that in the latter situation gene expression was induced by microbes present on the plant, not by *B. graminis* itself. It is possible that infection by *Z. tritici* may render the plant susceptible to saprophytes, endophytes or opportunistic microbes to which the plant then responds with characteristic defences. It is also possible that the microbial complement of the wheat host could alter responses to *Z. tritici* and thus affect the outcome of infection. Either process would result in a similar response in both compatible and incompatible interactions and might explain why different laboratories have reported differing results on the same pathosystem.

The genes and protein described here have well‐known roles in defence and senescence and are involved in responses to many other pathogens and abiotic stresses. Nevertheless, their expression during early phases of infection was not correlated with the compatibility or incompatibility of the interaction between wheat and *Z. tritici* genotypes, implying that other mechanisms or pathways, not necessarily involved in responses to better‐studied pathogens, must control genotype‐specific host responses to *Z. tritici*. A third conclusion, therefore, is that it is essential to investigate both compatible and incompatible interactions to understand the biology of host–parasite interaction in this complex pathosystem.

## Supporting information


**Figure S1.** Accumulation of TaMPK3 during compatible and incompatible interactions between wheat and *Zymoseptoria tritici*. Replicate gels showing changes in TaMPK levels over a 17 day period after inoculation with *Z. tritici* are shown by western blots probed with a TaMPK3‐specific antibody for eight different cultivar/isolate combinations: the interactions Longbow/IPO323, Avalon/IPO323 and Courtot/IPO88004 were compatible, the other combinations were incompatible. Protein loading levels are shown for each blot in the 60‐kD region using amido black staining. TaMPK3 accumulated in all cultivars inoculated with *Z. tritici*, independent of compatibility, some TaMPK3 also accumulated in the mock‐inoculated controls after 10 and 16 days.

**Figure S2.** Expression of six genes of interest where the replicates differed. Replicates 1–3 were inoculated with *Zymoseptoria tritici* isolate IPO323 and replicates 4–6 were inoculated with IPO88004. Relative expression of genes of interest was determined by qRT‐PCR compared with mock‐inoculated controls. All cultivar/isolate/time combinations have been combined for each replicate. Levels of expression of *PR1*,* peroxidase*,* β‐1*,*3‐glucanase*,* chlorophyll a/b binding precursor*,* chitinase* and *Mlo* differed between replicates. Significance of differences of the relative expression from 1 for each replicate: *** 0.001 >  *P*; ** 0.01 >  *P *>* *0.001; * 0.05 >  *P *>* *0.01.

**Figure S3.** Consistency of differential regulation between replicates. Expression of the genes of interest was determined by qRT‐PCR. With all cultivars combined for each gene, at each time point, the graph indicates whether the gene was up‐ or down‐regulated or no change was detected in each of six replicates (*y‐*axis).

**Figure S4.** Expression of the genes of interest during the interaction between wheat and *Zymoseptoria* between 0.5 and 14 days after inoculation. Expression of *β‐1*,*3‐glucanase chitinase*,* chlorophyll a/b binding precursor*,* cysteine protease* (*Sag12*), *lipoxygenase*,* Mlo*,* MPK3*,* PAL*,* PR1*,* peroxidase* and *PDI* was compared to mock‐inoculated controls at 0.5, 1, 3, 7, 10 and 14 days after inoculation by qRT‐PCR on eight cultivar/isolate combinations.
