# Supplementary material for: Early molecular signatures of responses of wheat to Zymoseptoria tritici in compatible and incompatible interactions
Source: Plant Pathol. 2016 Nov 22;66(3):450–9. doi: 10.1111/ppa.12633 (PMC5349288; doi:10.1111/ppa.12633)
Supplement: Supplementary file 1 — Figure S1. Accumulation of TaMPK3 during compatible and incompatible interactions between wheat and Zymoseptoria tritici. Replicate gels showing changes in TaMPK levels over a 17 day period after inoculation with Z. tritici are shown by western blots probed with a TaMPK3‐specific antibody for eight different cultivar/isolate combinations: the interactions Longbow/IPO323, Avalon/IPO323 and Courtot/IPO88004 were compatible, the other combinations were incompatible. Protein loading levels are shown for each blot in the 60‐kD region using amido black staining. TaMPK3 accumulated in all cultivars inoculated with Z. tritici, independent of compatibility, some TaMPK3 also accumulated in the mock‐inoculated controls after 10 and 16 days. [file PPA-66-450-s001.pdf]

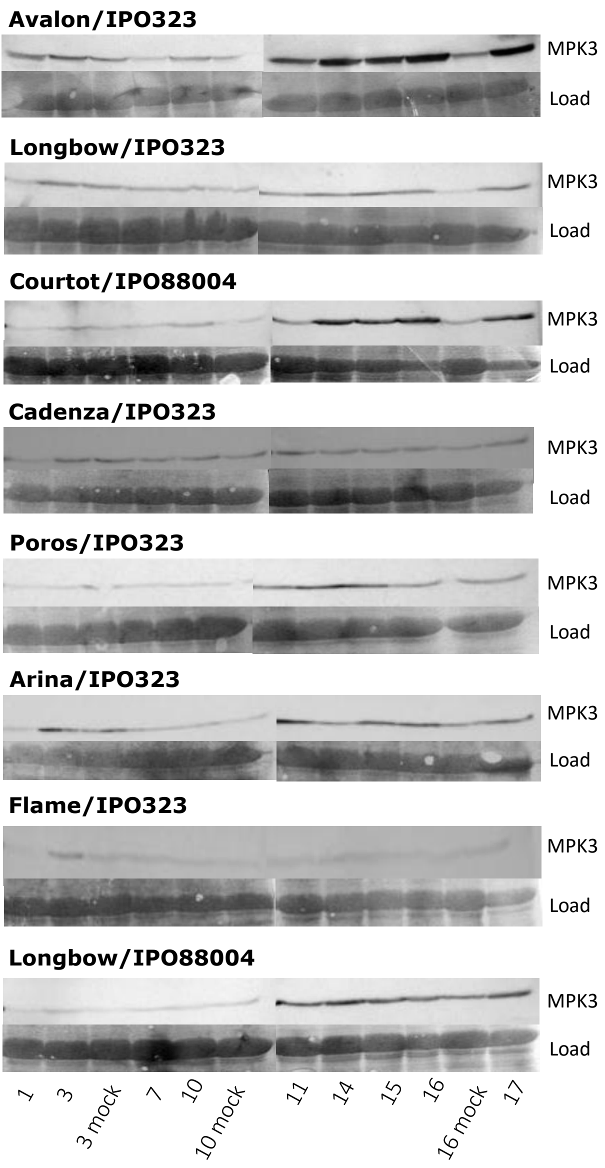

Figure S1. Accumulation of TaMPK3 during compatible and incompatible interactions between wheat and *Z. tritici*. Replicate gels showing changes in TaMPK levels over a 17 day period after inoculation with *Z. tritici* are shown by Western blots probed with a TaMPK3-specific antibody for eight different cultivar/isolate combinations: the interactions Longbow/IPO323, Avalon/IPO323 and Courtot/IPO88004 were compatible, the other combinations are incompatible. Protein loading levels are shown for each blot in the 60-kD region using amido black staining. TaMPK3 accumulates in all cultivars inoculated with *Z. tritici*, independent of compatibility, some TaMPK3 also accumulated in the mock-inoculated controls at 10 and 16 days.
