# Supplementary material for: Early molecular signatures of responses of wheat to Zymoseptoria tritici in compatible and incompatible interactions
Source: Plant Pathol. 2016 Nov 22;66(3):450–9. doi: 10.1111/ppa.12633 (PMC5349288; doi:10.1111/ppa.12633)
Supplement: Supplementary file 2 — Figure S2. Expression of six genes of interest where the replicates differed. Replicates 1–3 were inoculated with Zymoseptoria tritici isolate IPO323 and replicates 4–6 were inoculated with IPO88004. Relative expression of genes of interest was determined by qRT‐PCR compared with mock‐inoculated controls. All cultivar/isolate/time combinations have been combined for each replicate. Levels of expression of PR1, peroxidase, β‐1,3‐glucanase, chlorophyll a/b binding precursor, chitinase and Mlo differed between replicates. Significance of differences of the relative expression from 1 for each replicate: *** 0.001 > P; ** 0.01 > P > 0.001; * 0.05 > P > 0.01. [file PPA-66-450-s002.pdf]

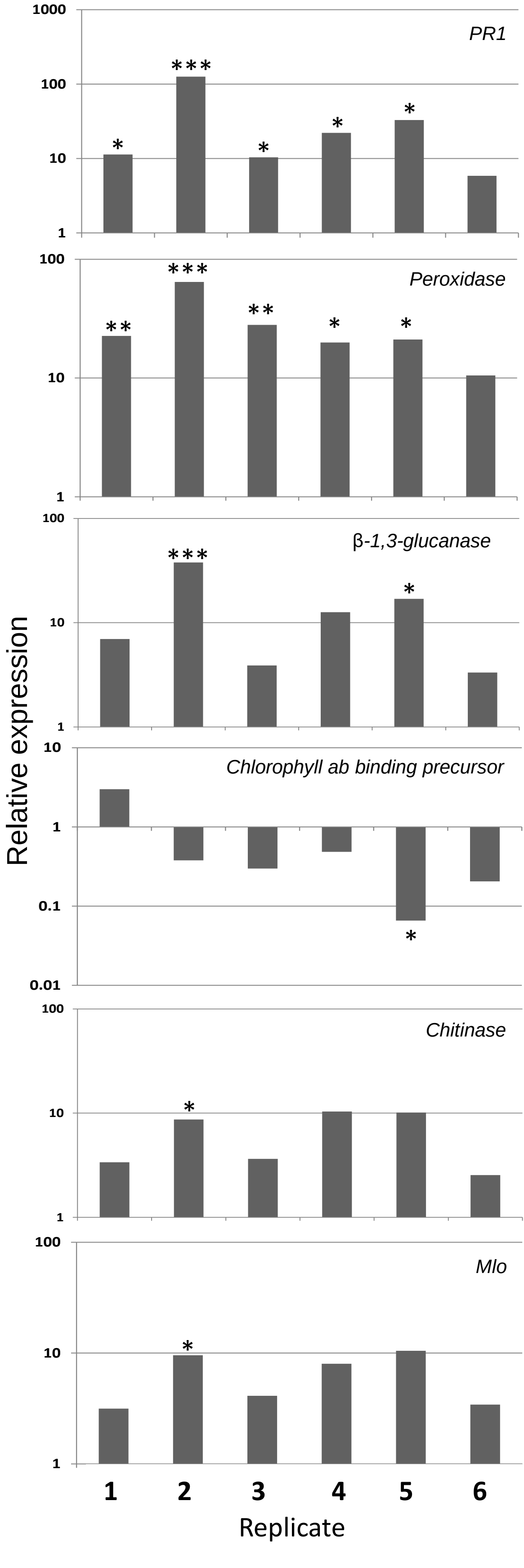

Figure S2. Expression of six genes of interest where the replicates differed. Replicates 1-3 are inoculated with *Z. tritici* isolate IPO323 and replicates 4-6 are inoculated with IPO88004. Relative expression of genes of interest were determined by RT-qPCR compared with mock-inoculated controls. All cultivar/isolate/time combinations have been combined for each replicate. Levels of expression of *PR1, peroxidase*, *β-1,3-glucanase*, *chlorophyll a/b binding precursor*, *chitinase* and *mlo* differed between replicates. Significance of differences of the relative expression from 1 for each replicate: *** 0.001>P; ** 0.01>P>0.001; * 0.05>P>0.01.
