# Supplementary material for: Early molecular signatures of responses of wheat to Zymoseptoria tritici in compatible and incompatible interactions
Source: Plant Pathol. 2016 Nov 22;66(3):450–9. doi: 10.1111/ppa.12633 (PMC5349288; doi:10.1111/ppa.12633)
Supplement: Supplementary file 3 — Figure S3. Consistency of differential regulation between replicates. Expression of the genes of interest was determined by qRT‐PCR. With all cultivars combined for each gene, at each time point, the graph indicates whether the gene was up‐ or down‐regulated or no change was detected in each of six replicates (y‐axis). [file PPA-66-450-s003.pdf]

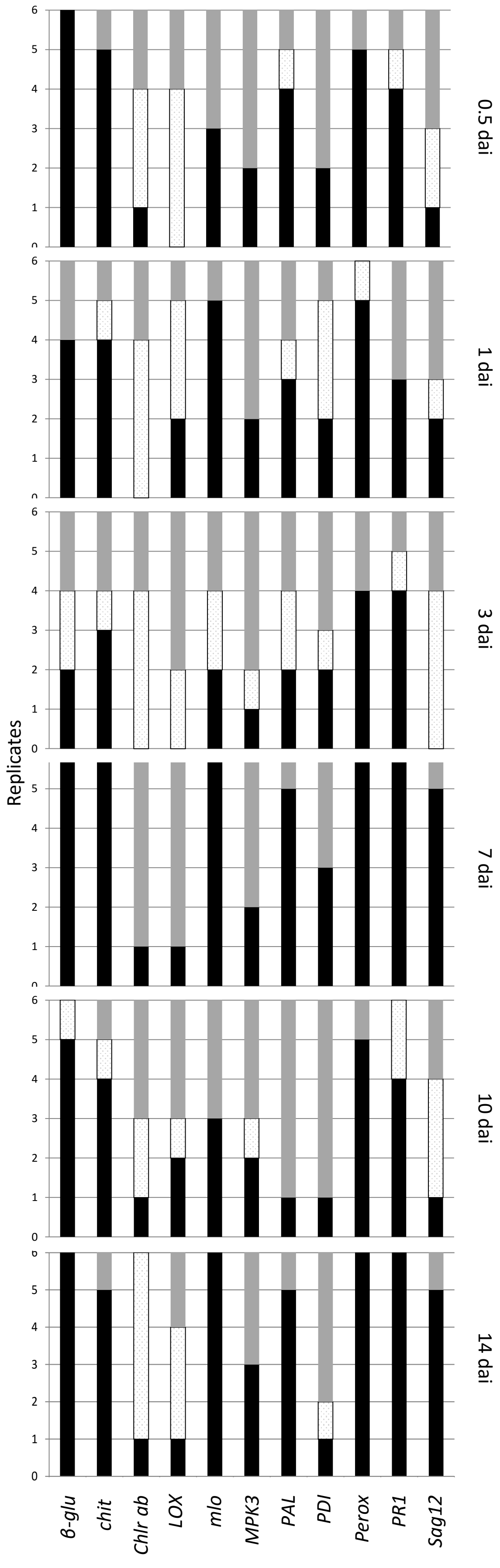

Figure S3. Consistency of differential regulation between replicates. Expression of the genes of interest was determined by RT-qPCR. With all cultivars combined For each gene, at each time point, the graph indicates whether the gene was, up- or down-regulated or no change was detected in each of 6 replicates (*y-axis*).
