# Supplementary material for: Early molecular signatures of responses of wheat to Zymoseptoria tritici in compatible and incompatible interactions
Source: Plant Pathol. 2016 Nov 22;66(3):450–9. doi: 10.1111/ppa.12633 (PMC5349288; doi:10.1111/ppa.12633)
Supplement: Supplementary file 4 — Figure S4. Expression of the genes of interest during the interaction between wheat and Zymoseptoria between 0.5 and 14 days after inoculation. Expression of β‐1,3‐glucanase chitinase, chlorophyll a/b binding precursor, cysteine protease (Sag12), lipoxygenase, Mlo, MPK3, PAL, PR1, peroxidase and PDI was compared to mock‐inoculated controls at 0.5, 1, 3, 7, 10 and 14 days after inoculation by qRT‐PCR on eight cultivar/isolate combinations. [file PPA-66-450-s004.pdf]

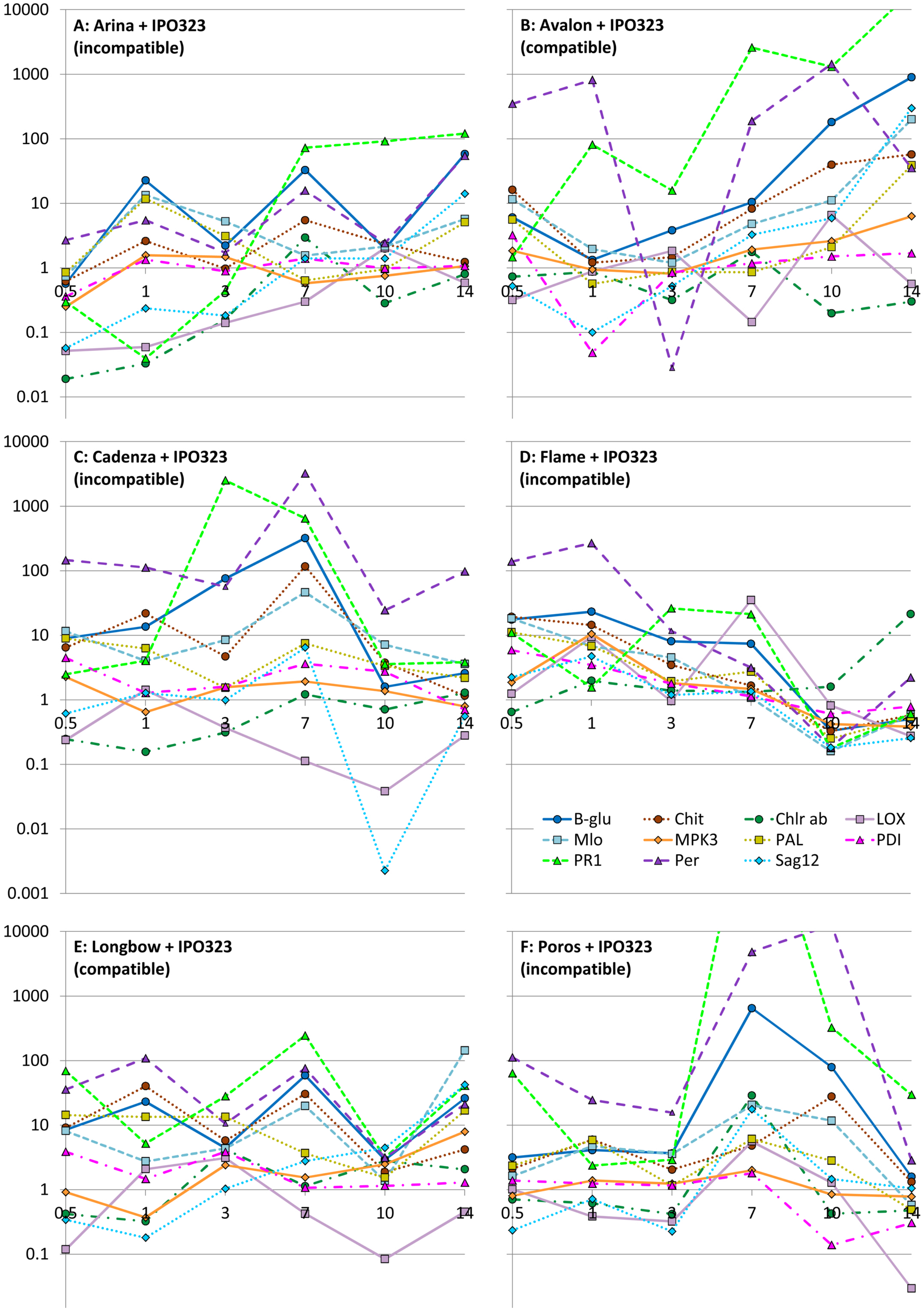

A: Arina + IPO323
(incompatible)
B: Avalon + IPO323
(compatible)
C: Cadenza + IPO323
(incompatible)
D: Flame + IPO323
(incompatible)
E: Longbow + IPO323
(compatible)
F: Poros + IPO323
(incompatible)
10000
1000
100
10
1
0.1
0.01
0.001
0.5
1
3
7
10
14
B-glu
Chit
Chlr ab
LOX
Mlo
MPK3
PAL
PDI
PR1
Per
Sag12

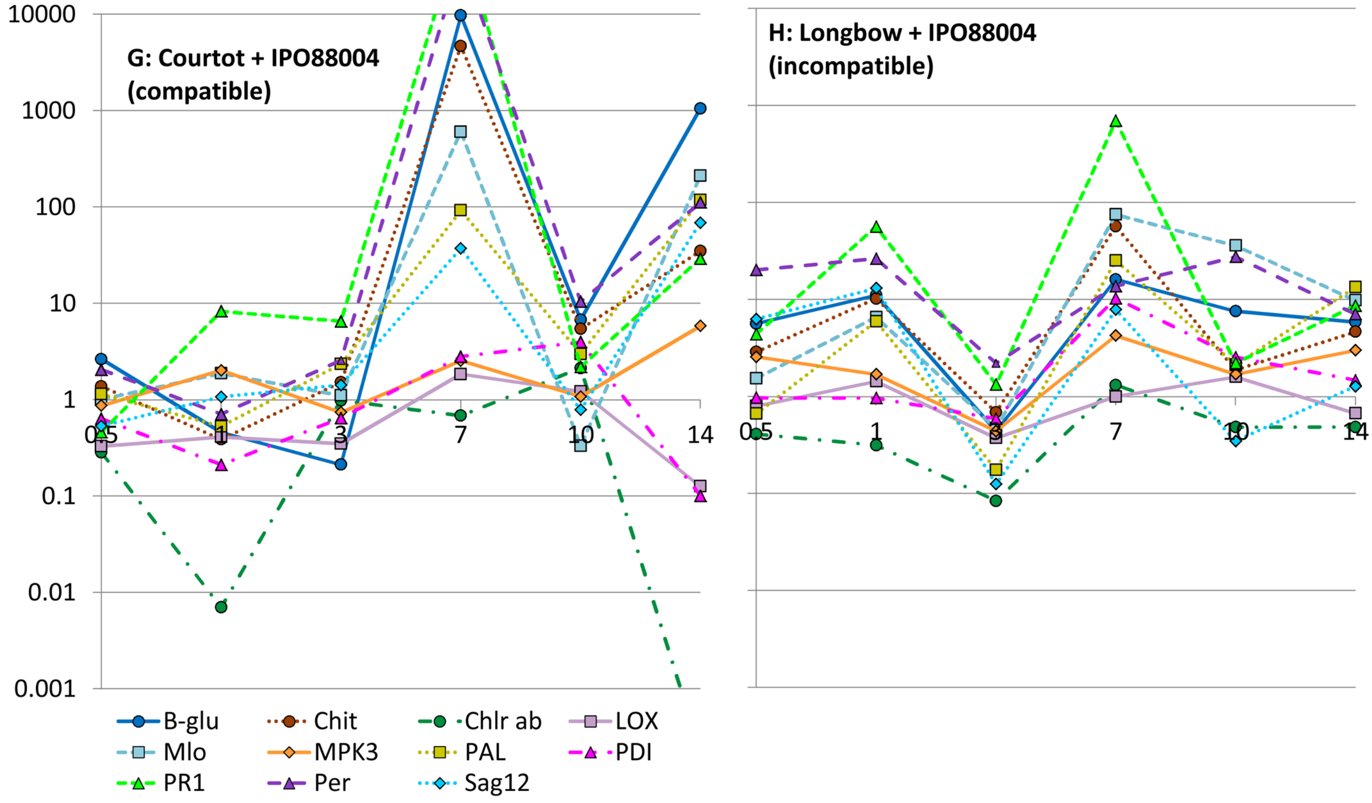

Supplementary Figure 4. Expression of the genes of interest during the interaction between wheat and *Zymoseptoria* between 0.5 and 14 days after inoculation. Expression of *β-1,3-glucanase*, c*hitinase, chlorophyll a/b binding precursor, cysteine protease* (*Sag12*), *lipoxygenase, mlo, MPK3, PAL, PR1, peroxidase* and *PDI* was compared to mock inoculated controls at 0.5, 1, 3, 7, 10 and 14 days after inoculation by qRT-PCR on eight cultivar/isolate combinations.
